# Supplementary material for: Interleukin 28B Polymorphism as a Predictor of Sustained Virological Response to Sofosbuvir-Based Therapy for Hepatitis C Virus Patients
Source: Trop Med Infect Dis. 2022 Sep 5;7(9):230. doi: 10.3390/tropicalmed7090230 (PMC9501239; doi:10.3390/tropicalmed7090230)
Supplement: Supplementary file 1 [file tropicalmed-07-00230-s001.zip › tropicalmed-1858568-supplementary.pdf]

Table S1. Correlation between PCR level of HCV RNA before treatment and demographic, laboratory, ultrasound of the liver and IL28b genotypes in non-responder group.

PCR of HCV RNA before treatment in non responder group / variables	Test of significance	*p*
**Age (years)**	0.194 [a]	0.160
**Gender**	0.225 [b]	0.102
**BMI (kg/m²)**	- 0.172 [a]	0.213
**WBCs(10³/cmm)**	0.214 [a]	0.119
**Haemoglobin(g/dl)**	0.057 [a]	0.681
**PLT(10³/cmm)**	-0.193 [a]	0.162
**INR**	0.010 [a]	0.945
**Total bilirubin (mg/dL)**	0.281 [a]	**0.039***
**ALT(IU/L)**	-0.231 [a]	0.092
**AST(IU/L)**	0.067 [a]	0.629
**Albumin (g/dL)**	0.080 [a]	0.566
**Creatinine (mg/dL)**	-0.197 [a]	0.153
**AFP**	0.200 [a]	0.147
**FIB-4**	0.242 [a]	0.078
**Ultrasound finding Of the liver**		
**Normal**		
**Parenchymatous liver disease**	P1=9.756 [d]	0.008*
**cirrhotic**	P2=-.830 [c]	0.407
	P3=-2.886 [c]	**0.004***
**IL28B genotype**		
	P4= 3.992 [d]	0.136
	P5= -1.864 [c]	0.062
	P6= -0.865 [c]	0.387
	P7= -1.098 [c]	0.272

(a) Pearson correlation, (b) Spearman correlation (c) Mann-Whitney, (d) Kruskall-Wallis, (P1) in between groups, (P2) between normal and both parenchymatous and cirrhotic groups, P3: between parenchymatous and cirrhotic groups. P4: in between all genotypes, P5 between TT and CT, P6 between CT and CC, P7 between TT and CC genotypes. *$p < 0.05$: statistically significant.

This study revealed statistically significance correlation between PCR level of HCV RNA before treatment and total bilirubin (p=0.039) and ultrasound finding of the liver (p=0.008) and no statistically significance correlation between it and age, gender, BMI, WBCs, haemoglobin, PLT, INR, ALT, AST Albumin, creatinine, AFP, Fib-4 and IL28B genotypes.
